# Manufacturing of Human Umbilical Cord Mesenchymal Stromal Cells on Microcarriers in a Dynamic System for Clinical Use

**DOI:** 10.1155/2016/4834616

**Published:** 2016-02-08

**Authors:** Florian Petry, J. Robert Smith, Jasmin Leber, Denise Salzig, Peter Czermak, Mark L. Weiss

**Affiliations:** ^1^Department of Anatomy and Physiology, Kansas State University, 228 Coles Hall, Manhattan, KS 66506, USA; ^2^Institute of Bioprocess Engineering and Pharmaceutical Technology, University of Applied Sciences Mittelhessen, Wiesenstraße 14, 35390 Gießen, Germany; ^3^Department of Chemical Engineering, Kansas State University, 1005 Durland Hall, Manhattan, KS 66506, USA; ^4^Faculty of Biology and Chemistry, Justus Liebig University, Ludwigstraße 23, 35390 Gießen, Germany; ^5^Fraunhofer Institute for Molecular Biology and Applied Ecology (IME), Project Group Bioresources, Winchester Straße 2, 35392 Gießen, Germany

## Abstract

The great properties of human mesenchymal stromal cells (hMSCs) make these cells an important tool in regenerative medicine. Because of the limitations of hMSCs derived from the bone marrow during isolation and expansion, hMSCs derived from the umbilical cord stroma are a great alternative to overcome these issues. For a large expansion of these cells, we performed a process transfer from static culture to a dynamic system. For this reason, a microcarrier selection out of five microcarrier types was made to achieve a suitable growth surface for the cells. The growth characteristics and metabolite consumption and production were used to compare the cells growth in 12-well plate and spinner flask. The goal to determine relevant process parameters to transfer the expansion process into a stirred tank bioreactor was achieved.

## 1. Introduction

Mesenchymal stromal cells (MSCs) play an important role in regenerative medicine, for cell therapy or tissue engineering [1–4]. This importance is based on properties of these cells. MSCs have the capacity to differentiate to osteoblasts, adipocytes, and chondrocytes, which classifies MSCs as multipotent stromal cells [5–8]. MSCs may modulate the immune system [8–14] and enable tissue repair [1] by secretion of growth factors, cytokines, and other signaling molecules into the medium [1, 15]. The immune properties of MSCs give these cells an important role to treat immunological disorders, such as graft-versus-host disease [9].

MSCs are found in other tissues beside the marrow cavity; for example, they can be found in blood or adipose tissue [8], dermis, muscle, dental pulp, umbilical cord blood, placenta, perivascular areas, amniotic fluid, and tissues surrounding the umbilical cord vessels, called Wharton's jelly [8, 9, 16]. The advantage of isolation of MSCs from the umbilical cord is that collection is safe and painless to mother and child, in contrast to the invasive and painful extraction of MSCs from the bone marrow.

While there are advantages to the choice of human umbilical cord mesenchymal stromal cells (hUC MSCs) as an MSC source, there are distinct challenges to using this source, which include the lack of standardized method for isolating, expanding, and validating hUC MSCs. These important limitations are not addressed here but are addressed in our companion paper [17].

The International Society for Cellular Therapy (ISCT) provides three minimal criteria to identify MSCs [18]. First, MSCs must be tissue culture plastic-adherent when maintained in standard culture conditions. Second, they express specific surface antigens CD105, CD73, and CD90 and they do not highly express markers of the hematopoietic lineage such as CD45, CD34, CD14, CD11b, CD79*α*, CD19, or HLA class II [18]. The third criterion is that MSCs must be capable of differentiating to osteoblasts, adipocytes, and chondrocytes* in vitro* [18]. Our group at the Kansas State University in Manhattan, Kansas [19–21], and two other laboratories, Dr. Davies' lab at the University of Toronto [22] and Dr. Fu at the National Yang-Ming University, Taipei [23, 24], have shown the isolation and characterization of hUC MSCs from Wharton's jelly and classified these cells as MSCs based upon their ability to produce bone, cartilage, and fat* in vitro* [8]. In addition to their differentiation capacity, hUC MSCs may differentiate to neuron-like cells [19, 25–28] and spontaneously beating cardiomyocytes [29]. Compared to hUC MSCs,* in vitro* expansion of adult bone-marrow-derived MSCs (BM MSCs) is slower [9]. When comparing the risks and safety margin, and the low cost and inexhaustible supply, hUC MSCs are a good alternative to BM MSCs to manage the graft-versus-host disease during the cell transplantation [9].

For a clinical use, large numbers of hUC MSCs (2-3 million MSCs·kg^−1^) and perhaps repeated doses are required [30–33]. The challenge of manufacturing the required amounts of cells requires a microcarrier-based stirred tank bioreactor process. The stirred tank bioreactor is a well-known, monitored, and controlled bioreactor system which enables a robust and reproducible culture process and a safe and reliable cell product according to Good Manufacturing Practice (GMP) and Good Clinical Practice (GCP) requirements. Since hUC MSCs are grown as adherent cells, suitable microcarriers must be chosen to provide an adequate growth surface.

Several research groups have shown MSC expansion in a dynamic bioreactor system. Chen et al. [34] show an optimized expansion of human fetal-derived MSCs in 2 L stirred tank cultures using Cytodex 3 microcarriers. They achieved a cell concentration of 1 × 10^6^ cells·mL^−1^ with confluent cell concentration of 4.7 × 10^4^ cells·cm^−2^. Dos Santos et al. [35] evaluated the expansion of BM MSCs and adipose tissue-derived MSCs (ASC). They developed a xenogeneic-free protocol for a 1 L-scale controlled stirred tank bioreactor with nonporous plastic microcarrier and analyzed different air concentrations in the medium and different medium exchanges and feeding strategies. They reached a final cell yield of (1.1 ± 0.1) × 10^5^ cells·mL^−1^ for the ASC MSCs and (4.5 ± 0.2) × 10^4^ cells·mL^−1^ for the BM MSCs. Cierpka et al. [36] reported the expansion of human MSCs derived from bone marrow in a disposable stirred tank bioreactor system according to GMP and PAT (Process Analytical Technology) requirements. The evaluated growth rates were 0.45 to 0.53 d^−1^; the final cell density was 5 × 10^4^ cells·cm^−2^ with a total cell number of 2.7 × 10^5^ cells·mL^−1^. The expansion was performed as a 2.4 L fed-batch process with a 6-times higher expansion factor compared to the batch cultivation. The growth surface was a collagen-coated microcarrier (SoloHill) and the culture was started with a low glucose DMEM medium and fed with a high glucose DMEM medium and fresh microcarriers. This expansion strategy, called bead-to-bead transfer, allows the low inoculation density of 800 cells·cm^−2^. The bead-to-bead transfer describes the migration of the cells from one microcarrier to another. It is assumed that during cell division the cells detach from one microcarrier and attach onto another microcarrier. The advantage of a bead-to-bead transfer is the prevention of large precultures.

Based upon this previous work, we considered a microcarrier-based process for the expansion of hUC MSCs in a high surface-to-volume ratio. Our goal was a robust and reproducible process according to GMP and GCP. To achieve this goal, this study focused on the characterization of the cell growth and metabolism of hUC MSCs and the process transfer from static culturing to a dynamic system. A small-scale spinner flask culture was used to identify the culture conditions for the expansion in the bioreactor. The crucial culture conditions we evaluated were the inoculation strategy, seeding density, stirrer speed, microcarrier type, and batch or fed-batch cultivation. We strived for a cultivation strategy with a high hUC MSC harvest yield which achieved all quality control criteria of these cells.

## 2. Materials and Methods

### 2.1. Umbilical Cord and Cell Isolation

Five human umbilical cords were used for this work. The cords were discarded tissues from apparently healthy, anonymous donors. The work with human tissues was reviewed by the Kansas State University Institutional Human Subjects Review Board and deemed to be not human subjects research (IRB review #5189). The MSCs were isolated from the umbilical cords using a method recently developed at Kansas State University [17]. For this study, we used cells at passage 3.

### 2.2. Culture Medium

The culture medium for hUC MSCs was based on DMEM low glucose (LG) medium (Gibco® by Thermo Fisher Scientific, Cat. number 11885-084) with addition of 10% pooled human platelet lysate (hPL, pooled from more than 25 expired units, obtained from Kansas University Medical Center, Dr. Lowell Tilzer, director), 1% GlutaMAX*™* (Gibco, Cat. number 35050-061), 1% Antibiotic-Antimycotic (Gibco, Cat. number 15240-062), and 0.4% Heparin (Kansas State University Veterinary Hospital Pharmacy, 1000 USP U·mL^−1^). All components were combined and sterile-filtered (0.22 *μ*m, Corning®, Cat. number 431098). The high glucose medium was made by the same procedure, but the DMEM LG medium is replaced by DMEM high glucose medium (Gibco, Cat. number 11995-065).

### 2.3. Growth Kinetic under Static Culture Conditions

The growth kinetic of five cell populations from different cords (two male donors, three female donors) in 2D was investigated in a 12-well plate (CytoOne, USA Scientific, Item number CC7682-7512) to determine the growth parameter and as a comparison to the cell growth in the dynamic system. The cells (passage 3) were seeded at a density of 8000 cells·cm^−2^ and a working volume of 1 mL DMEM LG medium. The cells were cultured for 7 days and harvested. To harvest, the medium of three wells was aspirated, washed once with 1 mL Dulbecco's phosphate buffered saline (DPBS), and lifted from the substrate with 0.05% trypsin-EDTA (Gibco, Cat. number 25200-056). The cell number, cell viability, and cell size were determined with the Cellometer Auto 2000 Cell Viability Counter using ViaStain AOPI staining kit (both from Nexcelom Bioscience, Waltham, MA).

### 2.4. Microcarrier Selection

For the expansion of hUC MSCs on microcarriers, five different microcarrier types (Animal Product-Free Starter Kit, SoloHill® by Pall Corporation) were compared (threefold determination, *n* = 3) to achieve the best growth and attachment conditions for the hUC MSCs. The starter kit contains Hillex® II, Plastic Plus, Plastic, Pronectin F-coated, and Glass-coated microcarriers (Table 1). In addition to these five microcarrier types, the effect of hydrogen chloride treatment on the Glass-coated microcarriers was tested. The Glass-coated microcarriers (100 g) were incubated in a bottle with 100 mL 1 M HCl overnight (16 h) at room temperature. After the incubation, the microcarriers were washed and dried. For each microcarrier type, 1 g microcarriers was autoclaved with 3 mL DPBS at 121°C and 100 kPa over pressure for 20 min. The microcarrier selection was performed in an ultralow attachment 6-well plate (Corning Costar®, Sigma-Aldrich®, Cat. number CLS3471-24EA) with a seeding density of 8000 cells·cm^−2^, 1.5 mL DMEM LG medium, and 25 g·L^−1^ microcarriers over 3 days for each microcarrier type. Every day, the cells of three wells were harvested. 1 mL was taken into a reagent cup, the microcarriers settled to the bottom, and we removed the supernatant, added 400 *μ*L of trypsin-EDTA (0.05%), and incubated the sample for 5 min under swirled conditions at 37°C and 5% CO_2_. The reaction was stopped with 800 *μ*L of hPL containing fresh and prewarmed culture medium and the cells were separated from the microcarrier through a cell strainer (Miltenyi Biotec, MACS SmartStrainers, 30 *μ*m, Cat. number 12-565-271). The cell strainer was rinsed three times with 1 mL DPBS. After centrifugation (5 min, 200 ×g), the supernatant was discarded and the cells were resuspended in fresh and prewarmed culture medium. Cell number, cell viability, and cell size were determined with the Cellometer Auto 2000 Cell Viability Counter.

### 2.5. Spinner Cultivation

The hUC MSCs were cultured in spinner flasks (Bellco, IOB W/MC FLASK, 100 mL, Cat. number 1965-61001) with 100 mL working volume and 25 g·L^−1^ microcarriers. The cells were seeded at 8000 cells·cm^−2^ and DMEM LG medium (see Section 2.2). For the first 24 h, the cells were cultured with 25 rpm stirrer speed to facilitate the attachment of the cells. After 24 h, the stirrer speed was increased to 40 rpm and every second day, the speed was increased by about 10 rpm to avoid microcarrier agglomeration. A 50% medium exchange was performed when the glucose concentration in the medium dropped to 0.2 g·L^−1^. The medium was exchanged with fresh and prewarmed culture medium with a glucose concentration of 2 g·L^−1^ to achieve an end concentration of 1.2 g·L^−1^ in the spinner flask. Every day a sample was taken from the spinner flask to determine cell number, cell viability, cell size, and metabolites (see Section 2.9). After 6–8 days of culture, the entire spinner was harvested. To harvest, the stirrer speed was set to 0 rpm to let the microcarriers settle down and as much as possible (80–90 mL) of the culture medium was removed. The microcarriers were washed two times with 20 mL prewarmed DPBS and incubated with 15 mL prewarmed 0.05% trypsin for 10 minutes at 37°C and 5% CO_2_ to detach the cells from the microcarriers. The trypsin reaction was stopped with 40 mL fresh and prewarmed culture medium. The cells were separated from the microcarriers through a cell strainer (30 *μ*m) and rinsed with DPBS. After centrifugation (5 min, 200 ×g), the supernatant was discarded and the cells were resuspended in fresh and prewarmed medium to determine cell number, cell viability, and cell size.

### 2.6. Differentiation Analysis

After spinner harvest, hUC MSCs were seeded in a 12-well plate (CytoOne, USA Scientific, Item number CC7682-7512) to confirm the differentiation capacity. This was performed according to the protocols of the StemPro® differentiation kits for adipogenesis, chondrogenesis, and osteogenesis by Thermo Fisher Scientific.

### 2.7. Cell Count and Maximal Growth Rate

Cell count, cell viability, and cell size were determined with the Cellometer Auto 2000 Cell Viability Counter using the ViaStain AOPI staining kit (both from Nexcelom Bioscience, Waltham, MA, USA). The exponential growth phase of the cells was estimated from a line created by plotting the natural logarithm of the cell number against the culture time. The slope of this linear curve is determined by linear regression analysis (GraphPad Prism, Version 5.01, GraphPad Software Inc.), which represents the maximal growth rate.

### 2.8. Microscopic Analysis of Cell Growth

The qualitative analysis was done by staining the cell nuclei on the microcarriers with SYBRGreen I (10,000x concentrate in DMSO, Cat. number S-7563). The stock solution of SYBRGreen I was diluted 1 : 10000 with DPBS for staining. For detection with a fluorescent microscope (EVOS® FL Auto Imaging System), an absorption wavelength of 497 nm and emission wavelength of 520 nm were used. The distribution and occupancy of microcarrier with cells were determined by counting the number of microcarriers with and without cells.

### 2.9. Metabolite Analysis

The metabolites glucose, glutamine, lactate, and ammonium were measured in the cell culture medium. The metabolite concentrations were measured using a BioProfile® 400 (Nova Biomedical, Waltham, MA). The consumption and production rates were determined by plotting the metabolite concentration over the culture time and calculation of the slope for linear consumption or production using linear regression analysis. The ratio of produced lactate to the consumed glucose called lactate yield and the ratio of produced ammonium to consumed glutamine (ammonium yield) for the static and dynamic cultivation were determined between 24 and 48 h. These yields serve for classification of the metabolism pathway.

### 2.10. Flow Cytometry

The flow cytometry methods employed here are identical to those described in our adjacent paper [17] to permit comparisons between the results of MSCs expanded in static culture and those expanded in dynamic culture. Briefly, the BD Stemflow*™* Human MSC Analysis Kit (BD Biosciences, Cat. number 562245) was used for positive and negative surface marker staining. Using the manufacturer's protocol, hUC MSC samples were stained with four fluorochromes together including positive and negative staining cocktails. The positive marker cocktail stained for CD90, CD105, and CD73 (defined as >97% positively stained cells). The negative cocktail (all antibodies were stained using a single fluorochrome, PE) stained for CD34, CD45, CD11b, CD19, and HLA-DR (defined as <2% positively stained cells). A CD44 labeled PE antibody was used as positive control for the negative cocktail to set the compensation and gating of the negative cocktail. For additional methodological details, see [17].

### 2.11. Colony Forming Unit-Fibroblast Assay

The CFU-F methods employed here are identical to those described in our adjacent paper [17] to permit comparisons between the results of hUC MSCs expanded in static culture and those expanded in dynamic culture. Briefly, hUC MSCs were plated at 5 or 10 cells·cm^−2^ in duplicates in 6-well CytoOne tissue culture plates in DMEM LG. Cells were expanded 4 days in culture, prior to fixation and methylene blue staining. For additional methodological details, see [17].

### 2.12. Karyotype Analysis

Following expansion of hUC MSCs in dynamic culture, the cells were cryopreserved to mimic how the cells might be banked prior to therapeutic application. The cells were subsequently thawed and plated at 1–1.5 × 10^4^ cells·cm^−2^ in DMEM LG, as described in [17]. Once attachment and expansion of the hUC MSCs were confirmed, the tissue culture flasks were submitted to Cell Line Genetics for karyotype analysis (Cell Line Genetics, Madison, WI).

## 3. Results

### 3.1. hUC MSC Growth Kinetic under Static Culture Conditions

**Figure 1:**
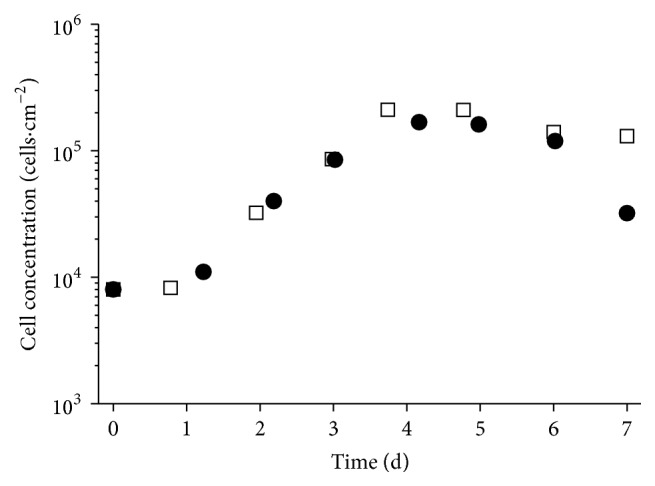
Growth kinetic of hUC MSCs from two different donors (HUC #255, female: white squares; HUC #256, male: black circles) in static 12-well culture plate in 1 mL DMEM LG medium with seeding density of 8000 cells·cm^−2^.

The growth kinetic in 12-well plates was done to identify differences in the cell growth and metabolism of hUC MSCs from five donors (Table 2) and serves as comparison for the dynamic system.  Figure 1 shows representative cell growth data of hUC MSCs from one male and one female donor. All cells were in passage number 3 for better comparability and to avoid any influence of senescence. No significant difference was observed between the cells from female or male donors. The exponential growth phase for all five cell types was observed between 20 and 100 h of culture time. The highest cell concentration at the end of the exponential growth phase reached values from 1.3 × 10^5^ to 2.1 × 10^5^ cells·cm^−2^. The mean value for the maximal growth rate *μ*_max_ was 0.042 ± 0.005 h^−1^ with a resulting doubling time of 16.8 ± 1.9 h and a mean fold expansion of 11.4 ± 0.7 after 7 days.

**Figure 2:**
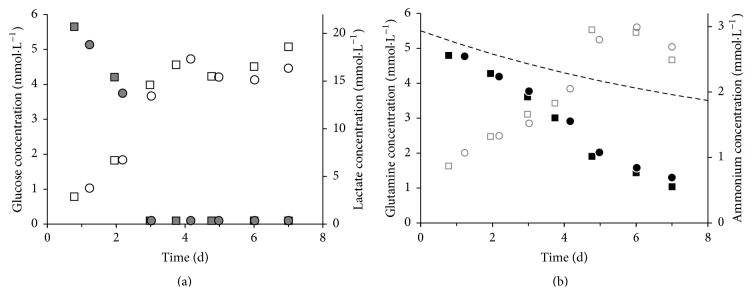
Metabolite profile of hUC MSCs from two different donors (HUC #255, female: squares, HUC #256, male: circles) in static 12-well culture plate in 1 mL DMEM LG medium with seeding density of 8000 cells·cm^−2^. (a) Grey full—glucose, white—lactate; (b) black—glutamine, grey empty—ammonium; dotted line: glutamine thermal decay.

The glucose-lactate (a) and glutamine-ammonium (b) profiles of hUC MSCs from a female and a male donor are shown in Figure 2. After 3 days the concentration of glucose in the medium was under the detection limit of 1.1 mmol·L^−1^ and the lactate concentration reached a constant level of 4.5 mmol·L^−1^ after 4 days. The glucose-lactate profile in relation to the cell growth (Figure 1) determined the glucose as limiting substrate for the hUC MSC proliferation. We determined for hUC MSCs from a female donor *Y*_Lac/Glc_ of 2.6 and for a male donor of 2.1 (see Supplemental Table  1 in the Supplementary Material available online at http://dx.doi.org/10.1155/2016/4834616). The consumption of glutamine was not limited and is connected with a constant production of ammonium. The ammonium yield *Y*_NH_4_/Gln_ of hUC MSCs from a female donor was 0.8 and from a male donor 0.5. A difference in the metabolism of hUC MSCs from male and female donors was not identified.

### 3.2. Microcarrier Selection

**Figure 3:**
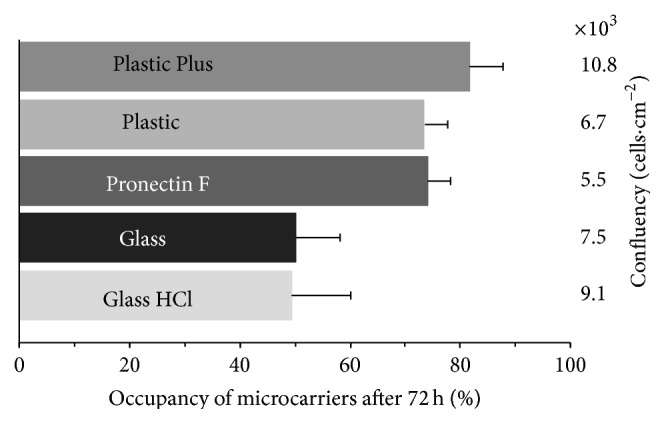
Occupancy and confluency of five different microcarriers after 72 h culture time in ultralow attachment 6-well plates with 0.0375 g of microcarriers in 1 mL DMEM LG medium at a seeding density of 8000 cells·cm^−2^. Bars represent one standard deviation of threefold determination.

**Figure 4:**
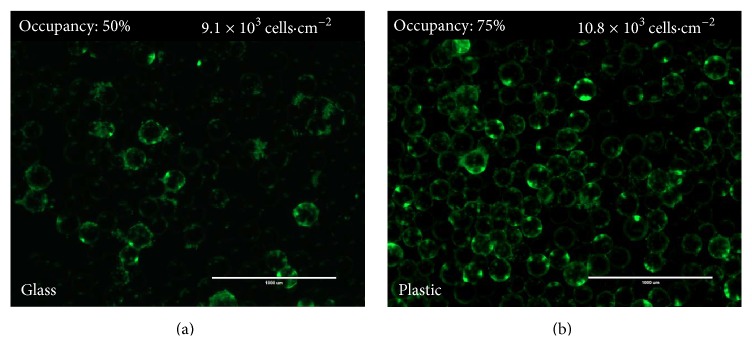
SYBRGreen stained hUC MSCs on HCl-treated Glass-coated microcarriers (a) and Plastic Plus microcarriers (b) after 3 days of culture in 1 mL DMEM medium in an ultralow attachment well plate at a seeding density of 8000 cells·cm^−2^ (scale bar = 1000 *μ*m).

The microcarriers play an important role for the expansion of adherent cells. For this reason, a microcarrier selection between five microcarrier types was done (Figure 3). The highest confluency and occupancy were the two crucial criteria for the selection of a suitable microcarrier type. The Pronectin F, Plastic, and Plastic Plus microcarriers showed a similar occupancy between 73 and 75%. The obtained cell number from the Pronectin F and Plastic was lower than that from the Plastic Plus microcarriers. Glass-coated microcarriers were investigated as untreated and pretreated with HCl. The pretreatment had a positive effect, which was visible in the higher final cell number. However, both Glass-coated microcarrier types showed a low microcarrier occupancy of around 50% (Figure 4). In consideration of the confluence and the occupancy, the Plastic Plus microcarriers were the best choice for the expansion of hUC MSCs.

### 3.3. hUC MSC Expansion Using Dynamic Spinner Cultivation

**Figure 5:**
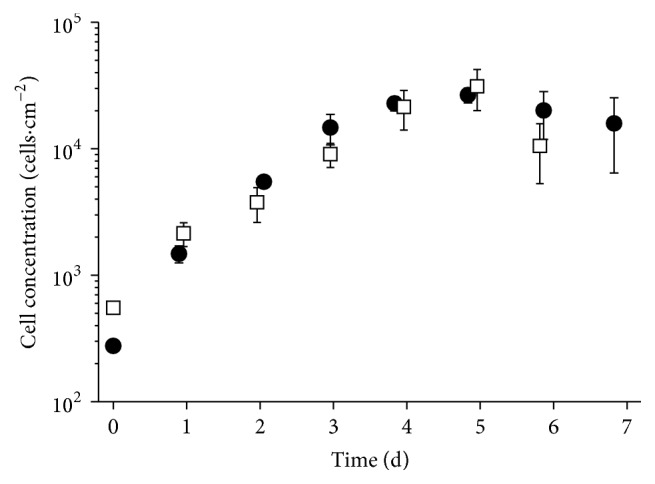
Growth kinetic of hUC MSCs on Plastic Plus microcarriers in spinner flasks in 100 mL DMEM LG medium with seeding density of 8000 cells·cm^−2^ (HUC #255, female: white squares; HUC #256, male: black circles).

Spinner flasks are a useful instrument for small-scale investigation of dynamic cell expansion. Inoculation methods with different resting times up to 4 h and occasionally briefly agitations were tested. There was no increase in cell growth or cell distribution based upon five independent tests (data not shown). We observed that inoculation strategies using agitation-rest-cycles led to an unwanted microcarrier agglomeration. Therefore we decided for an inoculation strategy under low (25 rpm) but continuous stirring. A seeding density of 8000 cells·cm^−2^ was found as suitable to avoid large precultures for inoculation and to prevent long culture times. To prevent microcarrier agglomeration with increasing cell number, the stirrer speed was increased every second day. The Plastic Plus and HCl-treated Glass-coated microcarriers were tested in nine spinner flasks trials. A nonhomogeneous cell distribution on the HCl-treated Glass-coated microcarriers was observed in the spinner flasks. Also the cell yield for HCl-treated Glass-coated microcarriers was lower compared to the Plastic Plus microcarriers (data not shown). We observed no difference in the growth between cells from male and female donors on Plastic Plus microcarriers (Table 3, Figure 5). In comparison to the static culture, the determined growth rates from the spinner flasks were comparable. The hUC MSCs could be efficiently detached with trypsin. An average cell yield of 4.2 × 10^7^  ± 1.4 × 10^7^ cells from an entire 100 mL spinner is related to a desired cell confluence of about 4.6 × 10^4^ cells·cm^−2^. This high efficiency of harvesting hUC MSCs was confirmed by observing bare microcarriers after SYBRGreen staining of the microcarriers following cell harvest.

**Figure 6:**
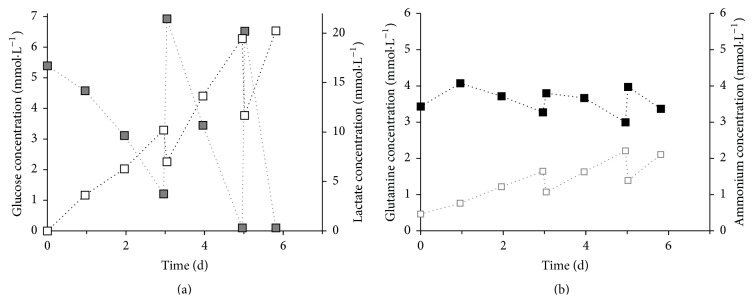
Metabolite profile of hUC MSCs from a female donor (HUC #255) in a dynamic spinner culture with 25 g·L^−1^, 100 mL DMEM LG medium, and a seeding density of 8000 cells·cm^−2^. (a) Grey full—glucose, white empty—lactate; (b) black full—glutamine, grey empty—ammonium.

With glucose identified as the limiting substrate, a 50% medium exchange was done before the glucose concentration dropped under 1.1 mmol·L^−1^.  Figure 6 shows a representative metabolite profile of hUC MSCs from a female donor in a spinner flask culture. The medium was exchanged after 3 and 5 days to achieve a desired glucose concentration of 5.5 mmol·L^−1^. We determined *Y*_Lac/Glc_ of 1.8 and *Y*_NH_4_/Gln_ of 1.3 between 24 and 48 h expansion time. The ground metabolism of hUC MSCs in spinner flasks cultures showed no difference between cells from a male and a female donor.

### 3.4. Bead-to-Bead Transfer

**Figure 7:**
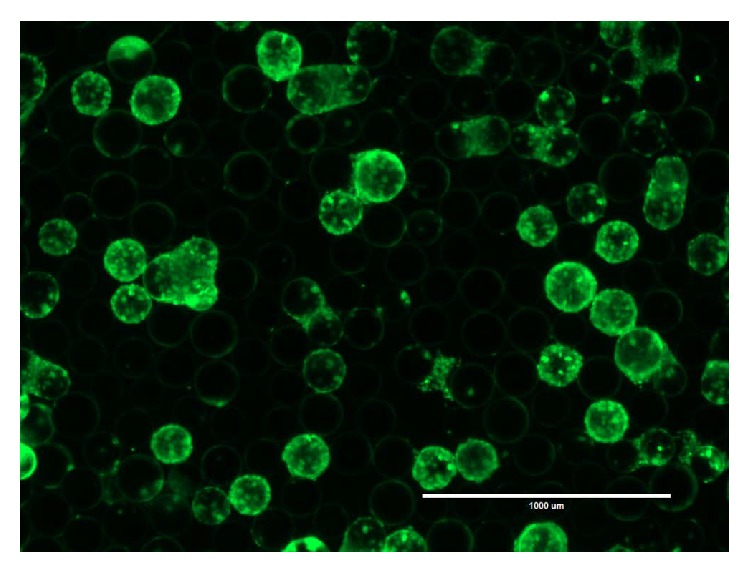
SYBRGreen stained hUC MSCs on Plastic Plus microcarriers after 9 days of culture in 200 mL DMEM medium in a spinner culture at a seeding density of 8000 cells·cm^−2^. The spinner culture was started with 100 mL working volume and 2.5 g microcarriers. After 4 days a feed of 100 mL fresh medium and 2.5 g fresh microcarriers was performed to investigate a bead-to-bead transfer. The microcarriers are either totally confluent with cells or blank (scale bar = 1000 *μ*m).

In our study, the bead-to-bead transfer was carried out with a feed of fresh medium and microcarriers after the first microcarriers were confluent. The working volume and microcarrier mass were doubled. The cells were cultured up to 10 days and a 50% medium exchange was performed, when the glucose concentration in the medium dropped under 1.1 mmol·L^−1^. As criterion for the bead-to-bead transfer, the cell distribution on the microcarriers was analyzed. After 2 to 3 days a slight increase of the occupancy to a constant level of about 60–70% was obtained. In contrast to the rapid rise of the occupancy over 80% after inoculation, the cells did not appear to migrate to fresh microcarriers. At the end of the culture period, the microcarriers were either fully confluent with cells or completely devoid of cells (blank) (Figure 7).

### 3.5. Quality Control of the hUC MSCs

**Figure 8:**
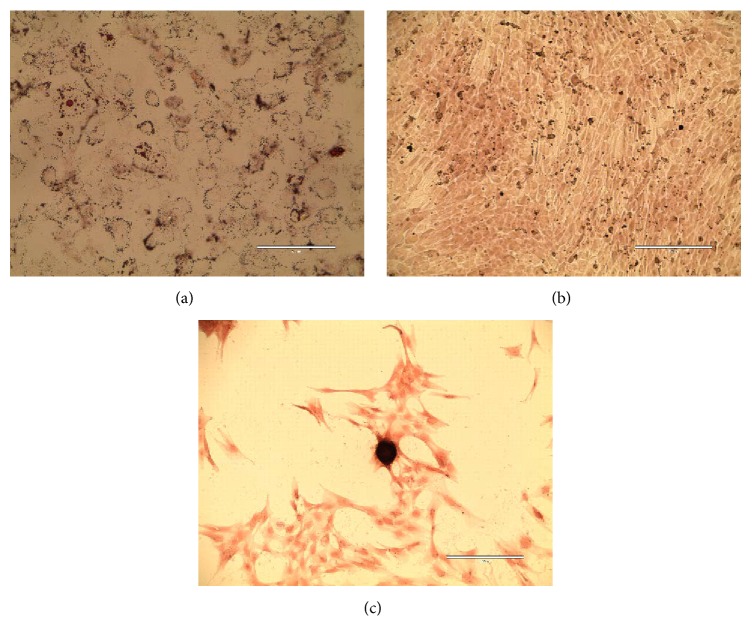
Differentiated hUC MSCs. (a) Adipocytes stained with Oil Red after 21 days (scale bar = 200 *μ*m); (b) osteocytes stained with Alizarin Red; and (c) chondrocytes stained with Safranin after 22 days (scale bar = 400 *μ*m).

After the harvest of the spinner flaks, the cells were seeded in 12-well plates to prove their ability to differentiate to adipocytes, chondrocytes, and osteoblasts. The dynamic expanded hUC MSCs were positive for lipid vesicle-forming adipocytes (Figure 8(a)), for calcium deposit-producing osteoblasts (Figure 8(b)), and nodule-forming chondrocytes (Figure 8(c)). Further, it was proven that the growth characteristics did not change after a spinner cultivation. We determined no change in the cell growth and a high cell viability over 90%. Flow cytometry analysis revealed that the 3D cultured hUC MSCs were positively stained for surface markers of MSCs and were negative for markers of the hematopoietic lineage (see Supplemental Figure  1). CFU-F analysis revealed that dynamically cultured MSCs had colony forming efficiency (CFE, defined as the number of MSCs plated divided by the number of colony forming unit-fibroblasts) of between 4.5 and 2.5 (see Supplemental Figure  2). Finally, after reviving hUC MSCs that had been previously expanded in dynamic culture, we found that 94% of the cells were viable at thaw. These cells were plated in static culture in 10% HPL enriched DMEM and they expanded robustly. The hUC MSCs were sent for karyotype analysis at passage 5, and the karyotype was normal (see Supplemental Figure  3). These results indicate that dynamically produced hUC MSCs meet ISCT MSC minimal definition.

## 4. Discussion

The goal was to investigate variables associated with the manufacture of hUC MSCs in a xeno-free, scalable, dynamic culture system. Three novel observations were made. First, after testing five different microcarriers with differing topography and surface chemistry, we found that Plastic Plus microcarriers were optimal for hUC MSC expansion. Second, after measuring medium metabolites during expansion, we found that glucose concentration was the critical variable for maintaining exponential MSC expansion. Third, by observing microcarrier occupancy, we determined that bead-to-bead transfer of MSCs does not occur to a significant degree. This is critical for designing scalable expansion end points.

Using the International Society of Cellular Therapy minimal definition, the hUC MSCs grown in static culture and in the xeno-free, dynamic cultivation met the minimal MSC definition [18]; this observation is in agreement with previous work of microcarrier-based expansion of MSCs [1, 2, 34–36] or more specifically hUC MSC [37]. Moreover, the colony forming efficiency of dynamically cultured hUC MSCs is comparable to that found in static culture [17]. Finally, a normal karyotype was found of hUC MSCs after microcarrier-based dynamic expansion, cryobanking, and expansion in static culture. These findings provide a basis for more highly refined microcarrier-based bioreactor experiments, scale-up, and validation studies. These results, together with our optimized hUC MSC isolation and xeno-free expansion work described in our adjacent paper [17], address critical information gap clinical manufacturing needs.

### 4.1. Comparison of hUC MSCs Expanded in Static and Dynamic Cultivation

In comparison to BM MSC the proliferation rate of hUC MSCs is higher, which results in lower doubling times [9, 38]. The determined max. growth rate of 0.042 ± 0.005 h^−1^ (*t*_*D*_ = 16.8 ± 1.9 h) is ~2-fold higher compared to the reported growth rates in the BM MSC literature. Majore et al. [39] determined a population doubling time of 27.5 ± 0.2 h for BM MSCs, which corresponds to a max. growth rate of 0.025 h^−1^, and Schop et al. [2] reported a growth rate of 0.020 ± 0.004 h^−1^ (*t*_*D*_ = 35.5 ± 6.00 h) for BM MSCs. The higher proliferation rate of hUC MSCs could be one explanation for the difference in the growth rates. Elseberg et al. [1] reported the influence of the glucose concentration in the medium on the cell growth. They used an immortalized cell line hMSC-TERT and reported a max. growth rate of 0.039 h^−1^ for the cells in low glucose medium (EMEM, 1 g·L^−1^ glucose) and a max. growth rate of 0.03 h^−1^ for the cells in high glucose medium (DMEM, 4.5 g·L^−1^ glucose). This shows the influence of the medium and the components on the MSC growth. In regard to the fact that every research group may use different medium formulations it is difficult to compare the cell growth between different laboratories. For this reason we performed the growth kinetic under static culture conditions to obtain our standard and as comparison for the dynamic system.

The challenge of the process transfer from static to dynamic system is the difference of the growth surfaces and the connected process of cell adhesion (static flat bottom to suspended round microcarrier). The higher cell density with sufficient nutrient supply and the potential harmful effect of shear forces created by stirring or aeration both may impact the proliferation of sensitive cells. The growth rate can be used as criterion to compare the different systems. The max. growth rate of 0.038 ± 0.008 h^−1^ in the spinner flask is comparable to the max. growth rate of 0.042 ± 0.005 h^−1^ from the 12-well plate and both systems show similar growth kinetics (Figures 1 and 5). Additionally, in both systems, no influence of the gender from the donor is observed. The final cell confluency of 5.9 × 10^4^ cells·cm^−2^ for the cultivation of the cells from a female donor is comparable with the reported concentrations from Chen et al. [34] and Cierpka et al. [36].

Glucose is the primary source for mammalian cells to generate ATP by oxidative phosphorylation or by anaerobic glycolysis [2]. *Y*_Lac/Glc_ indicates which metabolism pathway the cells prefer to use to produce energy [2, 40–42]. For the hUC MSCs in static culture we obtained *Y*_Lac/Glc_ of 2.6 (female) and 2.1 (male) and for the dynamic system *Y*_Lac/Glc_ of 1.8 (female and male). Schop et al. [2] declared that *Y*_Lac/Glc_ of 2 indicates that the cells use the inefficient glycolytic pathway instead of the oxidative phosphorylation to generate energy. The decrease of *Y*_Lac/Glc_ from static to dynamic hUC MSC expansion could be explained by changing the cellular metabolism from the glycolytic pathway to the oxidative phosphorylation by higher oxygen transfer in the spinner flask. The agitation in the spinner flask provides a homogenous distribution of substrates like glucose or oxygen in the culture medium, while the mass transfer in the static culture is driven by diffusion resulting in nutrient gradients.

Glutamine is the second main substrate for mammalian cell cultures and gets metabolically deaminated to glutamate [2]. We evaluated for the hUC MSCs *Y*_NH_4_/Gln_ of 0.8 (female) and *Y*_NH_4_/Gln_ of 0.5 (male) in the 12-well plates and *Y*_NH_4_/Gln_ of 1.3 (female) and 0.7 (male) for the dynamic culture. Schop et al. [2] gave *Y*_NH_4_/Gln_ of 1.6, but they provided reasons for not using the glutamine consumption and NH_3_ production for characterization: glutamine in the medium decays spontaneously and NH_3_ gets formed spontaneously. Assuming about ±10% glutamine decomposition per day [2, 43], Schop et al. calculated a glutamine consumption close to zero. Here we used 2 mmol GlutaMAX (glutamine dipeptide), which is a stabilized form of glutamine, and 4 mmol glutamine in our culture medium. NH_3_, besides being formed from glutamine decomposition, is also formed in the metabolism of several amino acids and the NH_3_ decreases by evaporation from the cultivation medium [2, 44]. These factors make it challenging to generate an accurate result about the glutamine metabolism of the cells. However, we calculated the daily 10% thermal glutamine decay in the static culture shown in Figure 2. The difference in the slope of the thermal decay and the curve of glutamine consumption by the hUC MSCs showed that the cells consumed the glutamine in the medium and the glutamine decrease cannot be reduced on the thermal decay. The glutamine consumption of hUC MSCs needs further investigations and, for this reason, the supply of the glutamine should be given only by GlutaMAX to differentiate between glutamine consumption by the cells and glutamine decay.

With increasing concentrations of lactate and ammonia, cell growth can be affected by a change of the pH and the cell toxic properties of these molecules. Schop et al. evaluated the toxic concentrations of lactate and ammonia for BM MSCs and found that cell growth decreased at a lactate concentration of 35.4 mmol and an ammonia concentration of 2.4 mM [2]. During the cultivation of the hUC MSCs in the 12-well plates these concentrations of lactate were never reached. After ±120 h the toxic levels of ammonia were exceeded in the 12-well plate, which could cause an increased cell death. On the other hand, we did not determine the toxicity of lactate or ammonia in hUC MSCs here, and it has not been reported in the literature to our knowledge. Due to the 50% medium exchange in the spinner flask it is likely that toxic levels of lactate and ammonium were not reached.

Five different hUC MSC isolates (3 female and 2 male) were expanded in static and dynamic cultivation. In the present study, no frank differences were observed between MSCs expanded in static or dynamic culture in differentiation capacity using the described qualitative differentiation assays. In contrast, Hupfeld et al. [37], who expanded hUC MSCs in static culture or dynamic culture using 10% fetal calf serum enriched medium, reported subtle differences in surface marker expression (static culture hUC MSCs produced with positive CD349 staining), gene expression, and cytokine secretion but did not report difference in differentiation potential between static and dynamic culture. In contrast, previous work by Goh et al. [45] and Tseng et al. [46] suggested that cultivation of MSCs on microcarriers may impact osteogenic differentiation potential, perhaps due to the shear stress that MSCs grown on microcarriers are subject to.

### 4.2. Microcarrier Selection and Bead-to-Bead Transfer

Cells derived from vertebrates have a heterogeneous negative charge on their surface. Suitable surfaces for the cell adhesion are dextran, glass, or plastic whose surface can be modified. During the adhesion process, electrostatic forces and van-der-Waals forces play an important role in the interaction of the cell and the growth surface. Divalent cations and glycoproteins from the medium are crucial factors for cell adhesion [47]. These factors show the importance of a suitable growth surface and have a major influence on cell proliferation. The higher occupancy of the Pronectin F, Plastic, and Plastic Plus microcarriers compared to the Glass-coated microcarriers (Figure 4) leads to the preliminary conclusion that an uneven microcarrier surface such as the cross-linked polystyrenes surface of these microcarriers provides more homogenous cell distribution and/or better cell attachment. The positive effect of the hydrogen chloride treatment on the Glass-coated microcarrier could occur through a modification of the microcarrier surface. The microcarrier surface may get charged or rougher (etched) like the modified cross-linked polystyrene surfaces of the other microcarriers, which could help the cells to attach. The acid may also remove the glass surface and exposes the plastic core of the microcarrier. These results support the conclusion that hUC MSCs prefer plastic as substrate for attachment and confirms that the Plastic Plus microcarriers were a good choice.

The bead-to-bead transfer was based on the assumption of cell detachment from the growth surface and reattachment following cell division. One reason for unsuccessful bead-to-bead transfer could be the ability of the cells to reattach on the microcarriers in a stirred system, but the cells showed this ability during the dynamic inoculation period. With this background the basic assumption of detachment and reattachment could be wrong, which covers the observation of Hu et al. [48]. For this reason, a homogenous distribution of the cells during the inoculation period is of great importance. However, Cierpka et al. [36] showed a successful bead-to-bead transfer with BM MSCs and collagen-coated microcarriers (SoloHill), which indicates the microcarrier as another source of error. We performed the microcarrier selection with the goal of a high cell yield and not a good bead-to-bead transfer. Perhaps there is another microcarrier type that combines both good cell growth and efficient bead-to-bead transfer and therefore is more suitable for this kind of hUC MSC expansion process. Presently, we do not know of such a microcarrier and this needs further investigation.

### 4.3. Influence of Donor and Source on Cell Proliferation

In our study we showed that the gender of the cell donor had no influence on the cell growth and metabolism. The mentioned difference in cell proliferation of MSCs derived from the bone marrow and umbilical cord [9, 38] could result from the relative age of the cells. MSCs derived from the umbilical cord are in fact fetal cells and compared to bone marrow MSCs which are usually isolated from adults. Also it is generally thought that stem cells with a low age have longer telomeres and have the capacity for extended expansion in culture and reduced senescent [8, 49]. Here, we did not evaluate whether hUC MSCs grown in dynamic culture maintain long telomeres. We previously described the long telomere length and telomerase expression of statically cultivated hUC MSC [19]. Flow cytometry determined that dynamically expanded hUC MSCs did not have different surface marker expression for the ISCT positive and negative surface marker set (see Supplemental Figure  1). This finding is in agreement with previous work by Hupfeld et al. [37] who found similar surface marker expression for UC MSCs for the ISCT marker set but different expression in CD349 (differentially expressed in statically cultured hUC MSCs and also adipose derived MSCs, but not those expanded in the bioreactor on microcarriers). Colony forming efficiency (CFE) of dynamically expanded UC MSCs was 4.5–2.5, which falls within the range observed for hUC MSCs expanded in static culture [17]. This indicates that UC MSCs maintain a high degree of self-renewal capacity following bioreactor based expansion and suggests that hUC MSCs remain stemmy or “young.” Finally, the karyotype of hUC MSCs expanded in dynamic culture and frozen and thawed was found to be normal. This indicates that MSCs subjected to the higher cytoskeletal stress (e.g., shear stress) of dynamic culture and freeze/thaw stress were genetically stable. Therefore, the procedures used for hUC MSC expansion may be safe for use in cellular therapy.

There is no limit in the age to donate BM MSCs, which is why the age of the cells from donor to donor can change strongly with range of many years. In addition, the bone marrow contents change over the lifespan. The red marrow space changes to a yellow marrow by fat deposition, which complicates the extraction [8]. Another aspect on the quality of the isolated cells could be the donor and the donor's physical health. The kind of lifestyle (healthy, unhealthy) or if the donor has/had any diseases could affect the condition on the cells. These aspects may have more impact on the cell proliferation and expansion than the gender of the donor.

## 5. Conclusion

We identified the relevant process parameters for a microcarrier-based expansion of hUC MSCs. To prevent microcarrier agglomeration a dynamic inoculation strategy and an increase of the agitation over the culture time are required. A microcarrier concentration of 25 g·L^−1^ and a seeding density of 8000 cells·cm^−2^ were suitable. The Plastic Plus microcarriers were suitable for the expansion of the hUC MSCs, but not for the bead-to-bead transfer.

A known problem of microcarrier-based expansion is the cell number determination. For this reason an online monitoring system like dielectric spectroscopy should be chosen for the expansion of hUC MSCs in a large-scale stirred tank bioreactor. The bead-to-bead transfer for hUC MSC expansion process should be investigated with other microcarrier types. For further experiments, a medium optimization towards a high glucose medium should be carried out to prevent a medium exchange. Another possibility is to establish a controlled and monitored glucose feed, with regard to the fact that the cell growth does not get affected by toxic levels of lactate and ammonium.

In the static and dynamic cultures of hUC MSCs, we found no difference in cell growth and metabolism between cells from male and female donors. On one hand this indicates a successful process transfer from static to dynamic system and on the other hand the age and source of the cells have more influence than the gender. In fact MSCs derived from umbilical cord are very young with a high proliferation activity and the advantages of cell isolation make these cells a good alternative to MSCs derived from bone marrow.

## Supplementary Material

Supplemental Figure 1: Surface marker analysis. Flow cytometry data represented in histograms, blue = test, red = isotype control. Negative (isotype control) gate was set to include 99% of the isotype; the positive gate percentages are shown for each sample. hUC MSCs were positive for A) CD90 stain B) CD105 stain C) CD73 stain, and negatively stained for the Negative cocktail (e.g., a mixture of CD34, CD45, CD11b, CD19, and HLA-DR). Note that CD44 was as a positive control (in black) for the negative cocktail and was used to set the negative control gate. E) CD 44 marker included as positive cocktail.
Supplemental Figure 2: Colony forming efficiency (CFE) at day 6 of incubation after plating at 5 or 10 cells∙cm^−2^. Data averaged from technical quadruplicates. CFE is defined as the number of plated cells divided by the number of colonies.
Supplemental Figure 3: Normal female karyotype was observed for MSCs passage 5 from HUC#255 following cultivation in spinner flask.
Supplemental Table 1: Metabolite Yields. Lactate and ammonium yields for hUC MSCs grown in static 12-well culture plates or dynamic spinner culture on microcarriers.

## Figures and Tables

**Table 1:** Microcarrier specification for Animal Product-Free Starter Kit by SoloHill, Pall Corporation.

Microcarrier type	Description	Relative density (g·cm^−3^)	Diameter (*µ*m)	Surface per gram (cm^2^·g^−1^)	Surface charge
Hillex II	Modified polystyrene	1.090–1.150	160–200	515	X

Pronectin F	Cross-linked polystyrene coated with recombinant RGD-containing protein	1.022–1.030	125–212	360	X

Plastic	Cross-linked polystyrene	1.022–1.031	125–213	360	

Plastic Plus	Cross-linked polystyrene, cationic charge	1.022–1.032	125–214	360	X

Glass	Cross-linked polystyrene coated with high silica glass	1.022–1.033	125–215	360	

**Table 2:** Maximal growth rate, minimum doubling time, and fold expansion of hUC MSCs from five donors, after 7 days in culture in 1 mL DMEM LG medium in a 12-well plate at a seeding density of 8000 cells·cm^−2^ (*n* = 3).

HUC #	Gender	Max. growth rate *µ*_max⁡_ (h^−1^)	Min. doubling time *t*_*D*_ (h)	Fold expansion
255	Female	0.045 ± 0.002	15.4 ± 0.6	12.7
260	Female	0.049 ± 0.002	18.1 ± 1.3	11.0
262	Female	0.037 ± 0.004	17.3 ± 1.7	10.9
256	Male	0.038 ± 0.003	14.2 ± 0.5	11.1
257	Male	0.040 ± 0.004	18.8 ± 2.1	11.2

Average		0.042	16.8	11.4
Standard deviation		0.005	1.9	0.7

**Table 3:** Growth characteristics for hUC MSCs on Plastic Plus microcarriers in spinner flasks with 100 mL DMEM LG culture medium at a seeding density of 8000 cells·cm^−2^.

Gender	Culture time (d)	Max. growth rate *µ*_max⁡_ (h^−1^)	Min. doubling time *t*_*D*_ (h)	Fold expansion	Highest occupancy (%)	Harvested cell yield
Male	7	0.046 ± 0.006	14.9 ± 2.2	16.4	81	2.6 × 10^7^
Female	6	0.032 ± 0.003	21.6 ± 2.5	13.8	93	5.3 × 10^7^
