# Supplementary material for: Manufacturing of Human Umbilical Cord Mesenchymal Stromal Cells on Microcarriers in a Dynamic System for Clinical Use
Source: Stem Cells Int. 2016 Feb 8;2016:4834616. doi: 10.1155/2016/4834616 (PMC4761675; doi:10.1155/2016/4834616)
Supplement: Supplementary file 1 — Supplemental Figure 1: Surface marker analysis. Flow cytometry data represented in histograms, blue = test, red = isotype control. Negative (isotype control) gate was set to include 99% of the isotype; the positive gate percentages are shown for each sample. hUC MSCs were positive for A) CD90 stain B) CD105 stain C) CD73 stain, and negatively stained for the Negative cocktail (e.g., a mixture of CD34, CD45, CD11b, CD19, and HLA-DR). Note that CD44 was as a positive control (in black) for the negative cocktail and was used to set the negative control gate. E) CD 44 marker included as positive cocktail. Supplemental Figure 2: Colony forming efficiency (CFE) at day 6 of incubation after plating at 5 or 10 cells∙cm−2. Data averaged from technical quadruplicates. CFE is defined as the number of plated cells divided by the number of colonies. Supplemental Figure 3: Normal female karyotype was observed for MSCs passage 5 from HUC#255 following cultivation in spinner flask. Supplemental Table 1: Metabolite Yields. Lactate and ammonium yields for hUC MSCs grown in static 12-well culture plates or dynamic spinner culture on microcarriers. [file 4834616.f1.zip › Description.docx]

Flow cytometry was used to analyze the surface marker expression in hUC MSCs following 3D expansion on Plastic Plus microcarriers in 10% HPL supplemented DMEM. High expression (>97% positive) for surface markers CD73, CD90, CD105 and CD44 was observed. Low surface maker expression (< 2% positive) was observed for CD34, CD45, CD11b, CD19, HLA-DR. A CD44 labeled PE antibody was used as positive control for the negative cocktail to set the compensation and gating of the negative cocktail. Additional details and comparable data from the 2D expansion are presented in our adjacent paper [17].

The hUC MSCs were plated after spinner cultivation at 5 or 10 cells⋅cm^−2^ in 6-well plates in DMEM LG for 4 days. Cells were fixed and stained with methylene blue. The CFU-F analysis revealed that dynamically cultured MSCs had colony forming efficiency (CFE) of between 4.5 and 2.5 and therefore comparable to the CFE of hUC MSCs only cultured in 2D [17].

Cytogenetic analysis was performed on 20 G-banded metaphase cells from hUC MSCs from spinner culture and all 20 cells demonstrated an apparently normal female karyotype and no non-clonal aberrations.

Figure legends:

**Supplemental Figure 1: Surface marker analysis.** Flow cytometry data represented in histograms, blue = test, red = isotype control. Negative (isotype control) gate was set to include 99% of the isotype; the positive gate percentages are shown for each sample. hUC MSCs were positive for A) CD90 stain B) CD105 stain C) CD73 stain, and negatively stained for the Negative cocktail (e.g., a mixture of CD34, CD45, CD11b, CD19, and HLA-DR). Note that CD44 was as a positive control (in black) for the negative cocktail and was used to set the negative control gate. E) CD 44 marker included as positive cocktail.

**Supplemental Figure 2:** Colony forming efficiency (CFE) at day 6 of incubation after plating at 5 or 10 cells∙cm^−2^. Data averaged from technical quadruplicates. CFE is defined as the number of plated cells divided by the number of colonies.

**Supplemental Figure 3:** Normal female karyotype was observed for MSCs passage 5 from HUC#255 following cultivation in spinner flask.

**Supplemental Table 1: Metabolite Yields**. Lactate and ammonium yields for hUC MSCs grown in static 12-well culture plates or dynamic spinner culture on microcarriers.
